# Supplementary figures and images for: PROZ Associated with Sorafenib Sensitivity May Serve as a Potential Target to Enhance the Efficacy of Combined Immunotherapy for Hepatocellular Carcinoma
Source: Genes (Basel). 2022 Aug 26;13(9):1535. doi: 10.3390/genes13091535 (PMC9498926; doi:10.3390/genes13091535)

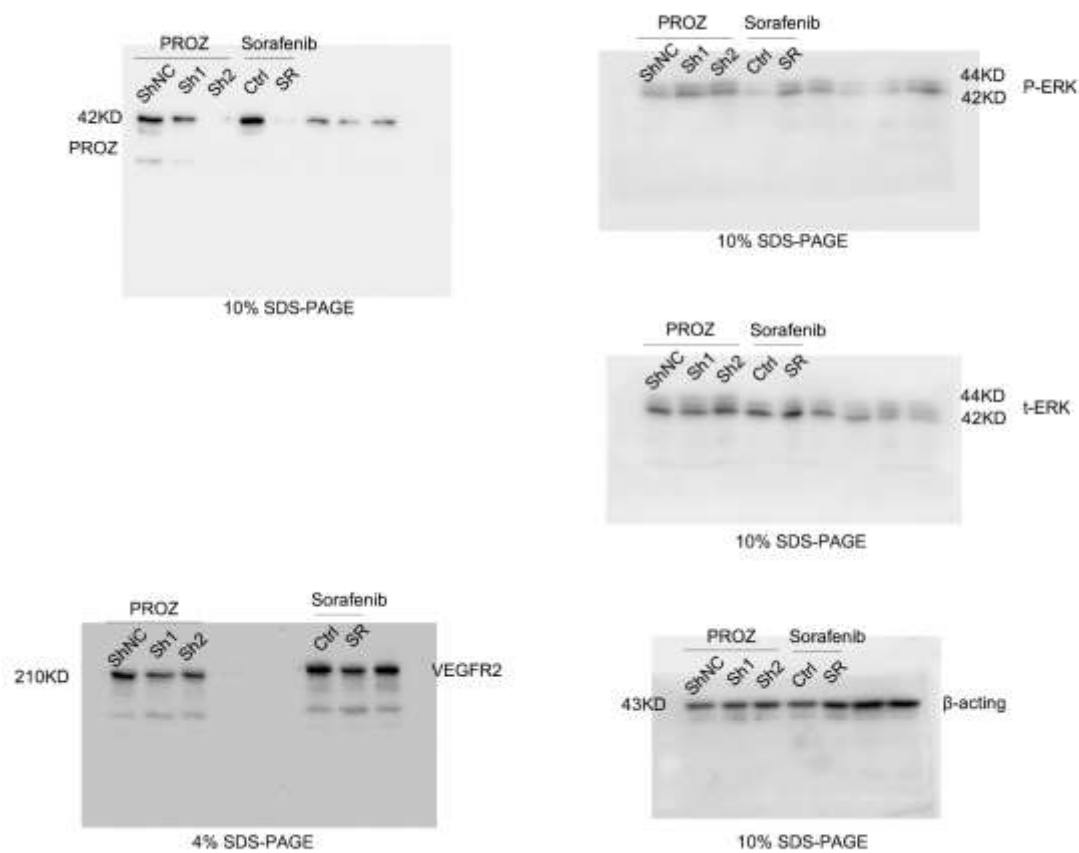

**Figure S1.** All bands and molecular weight markers of western blot.

Supplement: Supplementary file 1 [file genes-13-01535-s001.zip › genes-1855293-supplementary.pdf]
